# Reach and effectiveness of an HPV self-sampling intervention for cervical screening amongst under- or never-screened women in Toronto, Ontario Canada

**DOI:** 10.1186/s12905-023-02174-w

**Published:** 2023-01-25

**Authors:** Kimberly Devotta, Mandana Vahabi, Vijayshree Prakash, Aisha Lofters

**Affiliations:** 1 MAP Centre for Urban Health Solutions, St. Michael’s Hospital, Toronto, ON M5B 1T8 Canada; 2 Dalla Lana School of Public Health, University of Toronto, Toronto, ON M5T 3M7 Canada; 3 Daphne Cockwell School of Nursing, Toronto Metropolitan University (Formerly Ryerson University), Toronto, ON M5B 1Z5 Canada; 4 WECAN Research Project, Toronto Metropolitan University (Formerly Ryerson University), Toronto, ON M5B 1Z5 Canada; 5 Peter Gilgan Centre for Women’s Cancers, Women’s College Hospital, Toronto, ON M5S 1B2 Canada; 6 Department of Family and Community Medicine, University of Toronto, Toronto, ON M5G 1V7 Canada

**Keywords:** Cervical cancer, Cervical screening, HPV self-sampling, Community champions, Health equity, Immigrant health, RE-AIM

## Abstract

**Background:**

Cervical cancer is almost entirely preventable with appropriate and timely screening. In Ontario, Canada, South Asian, Middle Eastern and North African women have some of the lowest rates of screening and a suggested higher burden of cervical cancer. With increasing international evidence and adoption of HPV testing, many screening programs are making the move away from Pap tests and towards HPV testing with the option of HPV self-sampling seeming promising for under- or never-screened (UNS) women. Our study aimed to understand the uptake and acceptability of an HPV self-sampling intervention amongst these disproportionately UNS women in Peel region and surrounding areas in Ontario.

**Methods:**

A community -based mixed methods approach guided by the RE-AIM framework was used to recruit approximately 100 UNS racialized immigrant women aged 30–69, during the period of June 2018 to December 2019. The main recruitment strategy included community champions (i.e. trusted female members of communities) to engage people in our selected areas in Peel Region. Participants completed a study questionnaire about their knowledge, attitudes and practices around cervical cancer screening, self-selected whether to use the HPV self-sampling device and completed follow-up questions either about their experience with self-sampling or going to get a Pap test.

**Results:**

In total, 108 women participated in the study, with 69 opting to do self-sampling and 39 not. The majority of women followed through and used the device (n = 61) and found it ‘user friendly.’ The experience of some participants suggests that clearer instructions and/or more support once at home is needed. Survey and follow-up data suggest that privacy and comfort are common barriers for UNS women, and that self-sampling begins to address these concerns. Across both groups addressing misinformation and misconceptions is needed to convince some UNS women to be screened. Family, friends and peers also seemed to play a role in the decision-making process.

**Conclusions:**

HPV self-sampling is viewed as an acceptable alternative to a Pap test for cervical screening, by some but not all UNS women. This method begins to address some of the barriers that often prevent women from being screened and is already being offered in some jurisdictions as an alternative to clinical cervical cancer screening.

## Background

Appropriate and timely cervical screening can make the onset of cervical cancer almost entirely preventable. The impact of screening can be seen in Canada and other high-income countries where organized screening programs have decreased incidence and mortality in recent decades [1–4]. However, screening participation rates have shown some decline. In Ontario—Canada’s most diverse and populous province—rates of cervical screening have plateaued in the past two decades and have remained around 60% since 2013, well below provincial and national targets [5–7].

Previous work demonstrates that certain subgroups of women in Canada, including immigrants and women of low income, are at particular risk of under screening [8–35]. In Ontario, it has been shown that South Asian women are least likely to be screened, followed by Middle Eastern and North African women, where the adjusted odds ratio of screening for South Asian women compared to non-immigrant women was 0.61 (95% CI 0.59–0.64), and 0.68 (95% CI 0.64–0.72) for Middle Eastern and North African women [36]. These low levels of screening have been attributed to barriers such as not having a family physician, preference for a female physician, issues with transportation, cultural barriers (e.g. language barriers, different cultural norms around modesty) and indirect financial costs associated with screening (e.g., for childcare, taking time off work) [8–16, 21–25, 27–35]. The persistence of these low rates of screening suggest that innovative ways are needed to engage women and carry out cervical screening.

### Cervical cancer screening technology and HPV testing

The Ontario Cervical Screening Program (OCSP) was introduced in 2000 and recommends that everyone with a cervix commence cytology-based screening (i.e. a Papanicolaou test—‘Pap test’) at the age of 25 if they have been sexually active [37]. However, nearly all cases of cervical cancer are caused by Human Papillomavirus and increasing international evidence shows that HPV testing is more sensitive and accurate for detecting pre-cancers compared to Pap tests [38]. As a result, many areas around the world either have moved to HPV testing or are currently considering it for cervical cancer screening. In 2013 the OCSP and the Program in Evidence Based Care (PEBC)—an initiative of Cancer Care Ontario, the cancer agency arm of Ontario Health—recommended to Ontario’s Ministry of Health that HPV testing represented the best evidence-based approach for cervical cancer screening and that it was in fact the most accurate form of cervical screening [6, 38].

Additionally, unlike cytology-based screening, HPV testing provides an option for self-sampling devices that can be distributed in a number of ways, including take-home kits that can be mailed-in for testing. HPV self-samples are an accurate and usable cervical screening test [39]. The potential for these devices to help address cervical cancer under screening, is promising. In the United Kingdom, for example, the YouScreen study set out in 2021 to offer 31,000 people eligible for cervical screening in north and east London the opportunity to take a self-sample [40]. A study out of British Columbia, Canada that looked at self-sampling amongst under-housed women, found this method to be feasible for reaching women who do not receive routine cervical screening, and particularly effective for women who are at high-risk of cervical cancer [41]. Additional Canadian studies have also looked at the acceptability of HPV self-sampling, and have shown that it can significantly improve the participation of women who do not routinely attend organized screening programs [30] and is also an appropriate alternative for patients in low resource setting or reluctant to undergo pelvic examinations [41, 42]. In this study we were interested in understanding if HPV self-sampling is an effective intervention for UNS South Asian, West Asian, Middle Eastern and North African women living in the Greater Toronto Area in Ontario, Canada. Guided by the RE-AIM framework [43], we conducted an HPV self-sampling intervention study to understand the acceptability and uptake of self-sampling amongst these UNS women. We have previously published a detailed description of our protocol [44].

## Research question

In this paper we focus particularly on the reach (including describing the knowledge, attitudes and practices of women who participated) and effectiveness of the intervention.

## Methods

### Design

This study uses a community-based mixed methods design. Here we present the design and findings of our quantitative survey and follow-up.

### Target population

Informed by what were the current OCSP guidelines around cervical screening and the recommendations around HPV testing at the start of the study [2, 38], we defined our eligibility criteria to be: self-report of > 4 years since last Pap test (i.e. under screened) or no history of a Pap test (i.e. never screened); women aged 30–69 years; self-identifying as West or South Asian, Middle Eastern or North African; living in the Greater Toronto Area of Ontario, Canada; able to communicate in English and provide consent to participation. Women who had undergone a hysterectomy were included if they had retained their cervix. Women who had never been sexually active were excluded. Women who were pregnant were also excluded as the HPV Self-Sampling device that we used had not been trialed amongst pregnant women.

We used a targeted approach in the Peel Region as 51.5% of its population consist of immigrants, a large part of which are South Asian, as well as West Asian and Arab [45]. In Peel, South Asians are the largest visible minority population, making up 31.6% of the region’s population [45]. By contrast, across the province of Ontario, South Asians account for 8.7% of the overall population [45]. Punjabi and Urdu are the top two non-official home languages in Peel, with Tamil, Arabic and Gujarati amongst the top ten non-English and French languages [45].

### Recruitment strategy

Our recruitment strategy centred around the use of peers in the role of community champions (i.e. trusted female members of communities). In research, peers such as community champions can allow social access to participants and provide critical insight on how to make research a more comfortable and respectful experience for participants [46]. In our study, we considered a community champion to be a woman who identified as West or South Asian, Middle Eastern or North African and had pre-existing connections with local community groups and organizations in the Greater Toronto Area and Peel Region. The two study investigators (AL and MV) have done extensive work in the area of cancer screening for women who identify with these ethno cultural identities, and through previous work and word-of-mouth, we were able to identify community champions with proven knowledge and experience with these communities. We had a total of three community champions over the course of the study—one that was involved the entire duration of the study (VP) and two that worked in different capacities throughout the study. Since we were interested in recruiting women who had never been screened or were overdue, we knew that more traditional avenues of recruitment may not be representative of UNS women. For example, recruiting out of a healthcare centre or through healthcare providers would limit us to a sample of women who have at least some established level of access to healthcare. Our community champions recruited participants through various venues such as neighbourhood associations, places of worship, parent groups, cultural entertainment events, community organizers, tea parties and organizations with culturally-specific programming and mandates.

Recruitment activities consisted of distributing flyers and doing in-person presentations to groups of potential participants. During these interactions, a community champion and/or the study coordinator (KD; a South Asian woman herself) would introduce the study, explain the HPV self-sampling device and screen for eligibility. All potential participants were offered printed information materials from Cancer Care Ontario that explained cervical cancer, program eligibility and screening.

### Ethics

This study has received Research Ethics Board approvals from St. Michael’s Hospital (REB # 18-058; May 2018) and Toronto Metropolitan University, formerly Ryerson University (REB# 2018-219; June 2018). This study complies with the principles of the Declaration of Helsinki.

### Theoretical framework

Dissemination and Implementation (D&I) science is a growing area of research that aims to design interventions and identify implementation strategies that work in real life and across settings and populations [47]. RE-AIM (Reach, Effectiveness, Adoption, Implementation, Maintenance) is a D&I framework that can be used to assess how interventions have actually been implemented in practice. In particular, ‘Reach’ can help us to understand whose health or health behaviour will benefit from the intervention by looking at the absolute number, proportion and representativeness of people who are willing to participate in a given intervention, and their reasons why or why not [43, 48]. ‘Effectiveness’ helps us to identify which components of the intervention are considered necessary for the desired impact of the intervention; the impact of the intervention on important outcomes [43, 48].

### Intervention

Those who elected to try the HPV self-sampling kit were designated as Cohort A and those who did not were designated as Cohort B. Cohort A participants received their self-sampling kit in a postage paid return mailer box, either on the spot for in-person survey completion, or via mail for those that completed the survey over-the-phone. We used the HerSwab™ HPV self-sampling kit, a class 2 medical device approved by Health Canada (MDL license 94847). Eve Medical, the manufacturer of HerSwab, is an accredited ISO13485 medical device manufacturer. Throughout the study, women were mailed additional self-sampling kits if their original one was damaged or lost, or they needed to re-do their sample if the lab requested. All participants received a $30 honorarium, regardless of which cohort they were in. No additional compensation was given for completing the kit. Within a week of participation, the study coordinator followed up to see if the participant had tried the kit. If they had, they were then asked a series of questions on their experience using the kit. If they had not, they were given a reminder and asked when would be good to follow-up again. Follow-up calls continued until the kit was completed, they no longer wanted to participate or we exhausted all contact attempts. Participants in Cohort B were followed up with starting 3-months after they participated, to see if their interaction with the study had led them to get a Pap test or if they had plans to get a Pap test soon.

### Data collection

Once eligibility for the study was determined, participants then provided written consent and completed an interviewer-administered survey conducted by community champions or RC that asked demographics, and questions about knowledge, attitudes and practices, as well as questions around their decision to try or not try self-sampling. All interviews were done in-person or over the phone.

While conversational English was an eligibility criterion, the community champions were able to communicate in a variety of languages, to explain or interpret concepts that were difficult to comprehend in English. All participants also received a printed handout after survey completion with the correct answers to the knowledge questions that were in ‘true or false’ format, and more explanation on the statements that were presented in the attitudes section.

Recruitment took place from June 2018 to December 2019, with the last survey completed February 2020. Follow-up calls took place between June 2018 and January 2021. COVID-19 restrictions in March 2020 and onwards placed significant holds and challenges for community research and face-to-face interactions in Ontario, Canada. We had recruited all participants before restrictions were put in place, however, follow up efforts were impacted by limits that were placed by the research institution and provincial government. We also paused our follow up with Cohort B during times when provincial authorities had recommended a pause on cancer screening in Ontario.

We also conducted a qualitative portion of the study that consisted of five focus groups—3 with Cohort A and 2 with Cohort B—and interviews with key informants. The results of this portion will be presented elsewhere.

### Focus on ethnicity

Ethnicity refers to shared traits that are based on ancestry, social background, culture, tradition and language, and is based on self-identification [49]. Ethnicity is linked to health access and outcomes, and ethno cultural disparities have been seen in cancer screening in Ontario. We chose to focus on ethnicity—as reported by participants—as these subgroups of women have been identified as having particularly low rates of screening, and such an approach can allow us to tailor this intervention, and any future programming or services it informs, to socio-linguistic groups. Issues of ethnicity are particularly relevant in Canada, as over 200 different ethnic origins exist in the country [50]. Race/ethnicity is a social construct with very real implications for unequal power social relations and this is certainly seen in healthcare [51]. Race/ethnicity impacts how and if people access healthcare and the quality of care they receive. Paying particular attention to this in the design and evaluation of healthcare interventions, is to also recognize the different experiences and access to healthcare that people experience.

At the time of recruitment, we asked interested and potential participants: Do you identify as being of West or South Asian, Middle Eastern or North African background? Yes or No. If people were unsure, we showed them a list of countries from each of those regions. The naming of these regions is in line with how Statistics Canada collects and presents data, and is often used in popular media and government documents. We felt this approach would likely be the best understood by participants and community groups. Once consented and recruited, the study survey asked about self-identified ethnicity in the demographics section: How do you describe your ethnic/cultural origin? Participants were then presented with a list of countries that fall within West Asia, South Asia, the Middle East and North Africa. All participants were able to choose multiple responses if they self-identified with more than one response. As described in our recruitment and data collection methods, we took good care to use this focus on ethnicity, to inform our outreach (who recruited, where we recruited, what languages we used) and interviewing (setting, language assistance, approach to scheduling). Collecting data on ethnicity allowed us to more clearly describe the reach of our intervention.

### Data on reach and effectiveness

During recruitment, our community champions and study coordinator kept counts of the number of people that were: approached, interested and participated. Weekly recruitment reports tracked recruitment/outreach attempts and had counts of each of the community champion’s interactions. In the survey, we also collected demographics on: age, ethnicity, immigration, decade of arrival, relationship status, sexual orientation, number of children, English literacy, education, employment, and income. The main part of the survey asked participants to answer a series of validated ‘true or false’ questions about cervical cancer (knowledge), rate a series of statements on cervical screening (attitudes) and provide details on their Pap test history (practices). This information is used in this paper to describe and present the ‘reach’ of our intervention.

To understand effectiveness, we present data on the usage of the self-sampling kits, including the number of women that followed through with using and mailing it in, reported willingness to try it again and tested HPV positive. We also present data on their comments on the usability of the kit and preference compared to Pap tests. For Cohort B we present data from our follow up calls where we asked about Pap tests post-study participation.

### Data analysis

Data were analyzed using Statistical Package for the Social Sciences (SPSS) version 28. Descriptive statistics (e.g. frequency) summarized participants’ socio-demographic and cervical cancer knowledge, attitudes and practices. Survey and follow-up data was disaggregated by cohort.

## Results

### Reach

We recruited 108 participants for the study—69 who elected to try self-sampling (Cohort A) and 39 who did not (Cohort B). In total, our community champions and study coordinator interacted with 1645 people. This included people who attended a presentation about the study, approached our booth at events, took a flyer from us and interacted with us during recruitment. We tracked that 748 potential participants were interested and wanted to hear more. Of those that completed the screening questions, 202 were eligible and 452 were not eligible. Women who were eligible but declined to participate often cited concern for participating in research or needing to discuss participation with their family or healthcare provider. Others cited shyness and fear around discussing sexual health, cancer and previous screening experiences.

#### Demographics

Table 1 has a detailed breakdown of the collected demographics, by cohort. Of the women who participated in the study, the average age was 46 years in Cohort A and 45 years in Cohort B. The majority of our participants in both groups identified their ethnic/cultural identity as South Asian. Most women were from India or Pakistan (respectively, 49% and 30% in Cohort A, and 44% and 54% in Cohort B). Almost all of the participants were immigrants (i.e. Canadian citizen by naturalization, landed immigrant/permanent resident, refugee/refugee applicant), and most of them emigrated to Canada in the past two decades (Cohort A: 2000s—29% and 2010s—50%; Cohort B: 2000s—28% and 2010s—54%).

**Table 1:** Collected demographics of participants, by study cohort

	Cohort A (n = 69)	Cohort A %	Cohort B (n = 39)	Cohort B %	Total
*Ethnic/cultural origin*					
Afghan	5	7.2	0	0.0	5
Bangladeshi	2	2.9	0	0.0	2
Indian	34	49.3	17	43.6	51
Iranian	2	2.9	0	0.0	2
Pakistani	21	30.4	21	53.8	42
Sri Lankan	1	1.4	1	2.6	2
Iraqi	2	2.9	0	0.0	2
Turkish	1	1.4	0	0.0	1
Indo-Guyanese*	2	2.9	0	0.0	2
*Age (was collected as age)*					
30–39	23	33.3	18	46.2	41
40–49	23	33.3	7	17.9	30
50–59	14	20.3	6	15.4	20
60–69	8	11.6	7	17.9	15
Refused	1	1.4	1	2.6	2
*Citizenship and immigration*					
Canadian Citizen by birth	1	1.4	0	0.0	1
Canadian Citizen by naturalization	37	53.6	21	53.8	58
Landed immigrant/permanent resident	26	37.7	16	41.0	42
Refugee/refugee applicant	2	2.9	0	0.0	2
Other	3	4.3	2	5.1	5
*Decade of arrival (was collected as year) (n* = *107)*					
1970s	1	1.5	0	0	1
1980s	4	5.9	2	5.1	6
1990s	8	11.8	4	10.3	12
2000s	20	29.4	11	28.2	31
2010s	34	50.0	21	53.8	55
Refused	1	1.5	1	2.6	2
*Self-rated English literacy (i.e. reading, writing and speaking abilities)?*					
Excellent	23	33.3	16	41.0	39
Very good	16	23.2	10	25.6	26
Good	21	30.4	6	15.4	27
Fair	5	7.2	2	5.1	7
Poor	4	5.8	5	12.8	9
*What is your highest level of education?*					
Less than high school (grade 8 or less)	3	4.3	3	7.7	6
High School (12 grades) or equivalent	5	7.2	2	5.1	7
College (e.g. diploma) or university (e.g. BA, BSc) some or completed	26	37.7	24	61.5	50
Post-graduation (e.g. MA, PhD) some or completed	26	37.7	10	25.6	36
*Current employment status*					
Full-time employed (Minimum of 35 h/week)	19	27.5	7	17.9	26
Part-time employed	10	14.5	6	15.4	16
Unemployed	34	49.3	22	56.4	56
Other, please specify	6	8.7	4	10.3	10
*Approximate household annual income from all sources, after taxes*					
Less than $25,000	11	15.9	4	10.3	15
$25,000 to $40,000	6	8.7	4	10.3	10
$41,000 to $60,000	15	21.7	4	10.3	19
$61,000 to $75,000	9	13.0	5	12.8	14
More than $75,000	11	15.9	6	15.4	17
Other	8	11.6	4	10.3	12
Choose not to answer	9	13.0	12	30.8	21
*Marital status*					
Divorced/separated	6	8.7	2	5.1	8
Married/common law	56	81.2	35	89.7	91
Single, never married	1	1.4	2	5.1	3
Widowed	6	8.7	0	0.0	6
*Children*					
No	6	8.7	7	17.9	13
Yes	63	91.3	32	82.1	95

Number of children	Cohort A (n = 63)	Cohort A %	Cohort B (n = 32)	Cohort B %	Total
One	19	30.2	9	28.1	28
Two	22	34.9	11	34.4	33
Three	13	20.6	6	18.8	19
Four	6	9.5	3	9.4	9
Five	3	4.8	2	6.3	5
Nine	0	0.0	1	3.1	1

*People who are of Indian origin and have Guyanese nationality

Around 15% of participants (9% in Cohort A and 7% in Cohort B) rated their English literacy as either ‘fair’ or ‘poor’. Most of the participants had at least some post-secondary education (75% in Cohort A and 87% in Cohort B). About half of the participants in each cohort, were unemployed. Household incomes varied across both groups with a considerable proportion who refused to answer.

Most participants were married or in common law relationship, and everyone who responded to the question on sexual orientation (n = 107), identified as heterosexual. The majority of participants had children (91% in Cohort A and 82% in Cohort B), and most of them had one to three children.

#### Knowledge

In Table 2, we detail the ‘true or false’ responses to a series of 10 statements about cervical cancer screening. The majority of participants correctly responded to the question asking about the purpose of a Pap test (96% in cohort A and 90% in Cohort B). Similarly, most women across the two cohorts recognized the pap test should be performed regardless of having symptoms (e.g. vaginal infection or bleeding) (90% of those in Cohort A and 82% in Cohort B).

**Table 2:** Responses to knowledge questions by study cohort (italicized column headings indicate the correct response)

	Cervical cancer can be prevented			Cervical cancer can be cured			Cervical cancer can have no signs in its early stages			Having a Pap test may help detect cervical cancer earlier			A woman's first Pap test should be done at age 21, whether or not she has been sexually active		
	Don't know	FALSE	*TRUE*	Don't know	FALSE	*TRUE*	Don't know	FALSE	*TRUE*	Don't know	FALSE	*TRUE*	Don't know	*FALSE*	TRUE
Cohort A (n = 69)	13	3	*53*	15	2	*52*	20	11	*38*	2	1	*66*	20	*23*	26
Cohort B (n = 39)	7	1	*31*	10	0	*29*	11	9	*19*	2	2	*35*	7	*10*	22

	Women should have a Pap test every year			The Pap test is recommended only for women who have been sexually active			The Pap test should be performed only if a woman has a vaginal infection or bleeding or other worrisome symptoms			The Pap test can be done among pregnant women			The Pap test may cause vaginal infection		
	Don't know	*FALSE*	TRUE	Don't know	FALSE	*TRUE*	Don't know	*FALSE*	TRUE	Don't know	FALSE	*TRUE*	Don't know	*FALSE*	TRUE
Cohort A (n = 69)	8	*39*	22	7	34	*28*	3	*62*	4	11	37	*21*	17	*44*	8
Cohort B (n = 39)	5	*27*	7	6	21	*12*	3	*32*	4	6	20	*13*	7	*22*	10

	Cervical cancer can be prevented			Cervical cancer can be cured			Cervical cancer can have no signs in its early stages			Having a Pap test may help detect cervical cancer earlier			A woman's first Pap test should be done at age 21, whether or not she has been sexually active		
	Don't know	FALSE	*TRUE*	Don't know	FALSE	*TRUE*	Don't know	FALSE	*TRUE*	Don't know	FALSE	*TRUE*	Don't know	*FALSE*	TRUE
Cohort A %	18.8	4.3	*76.8*	21.7	2.9	*75.4*	29.0	15.9	*55.1*	2.9	1.4	*95.7*	29.0	*33.3*	37.7
Cohort B %	17.9	2.6	*79.5*	25.6	0.0	*74.4*	28.2	23.1	*48.7*	5.1	5.1	*89.7*	17.9	*25.6*	56.4

	Women should have a Pap test every year			The Pap test is recommended only for women who have been sexually active			The Pap test should be performed only if a woman has a vaginal infection or bleeding or other worrisome symptoms			The Pap test can be done among pregnant women			The Pap test may cause vaginal infection		
	Don't know	*FALSE*	TRUE	Don't know	FALSE	*TRUE*	Don't know	*FALSE*	TRUE	Don't know	FALSE	*TRUE*	Don't know	*FALSE*	TRUE
Cohort A %	11.6	*56.5*	31.9	10.1	49.3	*40.6*	4.3	*89.9*	5.8	15.9	53.6	*30.4*	24.6	*63.8*	11.6
Cohort B %	12.8	*69.2*	17.9	15.4	53.8	*30.8*	7.7	*82.1*	10.3	15.4	51.3	*33.3*	17.9	*56.4*	25.6

Participants, and in particular those in Cohort B, were most incorrect about the statements related to Pap test initiation, intervals and eligibility. ‘A woman's first Pap test should be done at age 21, whether or not she has been sexually active’, was considered ‘false’ by only 26% of participants in Cohort B. Slightly more people (33%) knew the statement to be false in Cohort A. In Cohort B, only 31% of participants knew that the Pap test is recommended only for women who have been sexually active (vs. 41% in Cohort A). More people in Cohort B correctly responded ‘false’ to ‘Women should have a Pap test every year’, with 69% in Cohort B and 57% in Cohort A, responding so.

#### Attitudes

Participants were also asked to indicate their level of agreement on a five-point scale with a series of statements about Pap tests (Table 3). In both groups, most participants believed ‘having a Pap test lowers my chances of getting cervical cancer’ with 81% of Cohort A and 85% of Cohort B indicating they either ‘completely agreed’ or ‘agreed.’ In Cohort A, 40% of participants ‘completely agreed’ or ‘agreed’ that a Pap test was painful, and in Cohort B it was 51%. Somewhat similar numbers were also seen amongst participants when presented with the statement ‘Performing the Pap test invades a woman’s privacy’, where 41% in Cohort A and 31% in Cohort B ‘completely agreed’ or ‘agreed’. Time was considered to be an issue for some, as 36% in Cohort A and 28% in Cohort B felt the Pap test was time consuming.

**Table 3:** Responses to attitudes questions, by study cohort

	Getting a Pap test is expensive					The Pap test is painful					Getting a Pap test is time consuming				
	Completely Agree	Agree	Disagree	Completely disagree	No idea	Completely Agree	Agree	Disagree	Completely disagree	No idea	Completely Agree	Agree	Disagree	Completely disagree	No idea
Cohort A	1	4	36	16	12	6	22	24	7	10	6	19	28	10	6
Cohort B	4	1	12	17	5	7	13	10	6	3	4	7	17	8	3

	Performing the Pap test invades a woman's privacy					Having a Pap test lowers my chances of getting cervical cancer					The equipment used to do the Pap test is not of good quality				
	Completely Agree	Agree	Disagree	Completely disagree	No idea	Completely Agree	Agree	Disagree	Completely disagree	No idea	Completely Agree	Agree	Disagree	Completely disagree	No idea
Cohort A	1	27	5	14	20	23	33	7	1	5	0	6	32	7	24
Cohort B	1	11	5	10	9	19	14	2	1	3	3	3	13	10	10

	Getting a Pap test is expensive					The Pap test is painful					Getting a Pap test is time consuming				
	Completely Agree	Agree	Disagree	Completely disagree	No idea	Completely Agree	Agree	Disagree	Completely disagree	No idea	Completely Agree	Agree	Disagree	Completely disagree	No idea
Cohort A	1.4	5.8	52.2	23.2	17.4	8.7	31.9	34.8	10.1	14.5	8.7	27.5	40.6	14.5	8.7
Cohort B	10.3	2.6	30.8	43.6	12.8	17.9	33.3	25.6	15.4	7.7	10.3	17.9	43.6	20.5	7.7

	Performing the Pap test invades a woman's privacy					Having a Pap test lowers my chances of getting cervical cancer					The equipment used to do the Pap test is not of good quality				
	Completely Agree	Agree	Disagree	Completely disagree	No idea	Completely Agree	Agree	Disagree	Completely disagree	No idea	Completely Agree	Agree	Disagree	Completely disagree	No idea
Cohort A	1.4	39.1	7.2	20.3	29.0	33.3	47.8	10.1	1.4	7.2	0.0	8.7	46.4	10.1	34.8
Cohort B	2.6	28.2	12.8	25.6	23.1	48.7	35.9	5.1	2.6	7.7	7.7	7.7	33.3	25.6	25.6

#### Practices

Table 4 breaks down the Pap test history of participants in the study. Around 70% of women in each group, had done a Pap test before. In both groups, around three-quarters (75%) of women had their most recent Pap test 4–7 years ago.

**Table 4:** Pap test histories and practices, by study cohort

	Cohort A (n = 69)	Cohort A %	Cohort B (n = 39)	Cohort B %	Total
*Had a Pap test before*					
No	19	27.5	12	30.8	31
Yes	49	71.0	27	69.2	76
Unsure	1	1.4	0	0.0	1

	Cohort A (n = 49)	Cohort A %	Cohort B (n = 27)	Cohort B %	Total
*Time since last Pap test*					
4 years ago	14	28.6	8	29.6	22
4+ to 7 years ago	25	51.0	12	44.4	37
8 to 10 years ago	6	12.2	5	18.5	11
More than 10 years ago	4	8.2	2	7.4	6

Why have you not done a Pap test within the past 4 years? Choose as many as apply—Selected Choice			
	Cohort A	Cohort B	Total
Cost	1	0	1
Didn't think it was necessary	14	10	24
Don't know what a Pap test is	0	3	3
Fear (e.g. of pain, of embarrassment)	14	6	20
Female provider not available to do the test	6	2	8
Had personal or family responsibilities	19	8	27
My doctor didn't think it was necessary	6	1	7
Transportation problems	2	0	2
Wait time was too long	5	1	6
Other, please specify	20	11	31

Why have you never had a Pap test? Choose as many as apply—Selected Choice			
	Cohort A	Cohort B	Total
Cost	0	1	1
Didn't think it was necessary	9	7	16
Don't know what a Pap test is	11	5	16
Fear (e.g. of pain, of embarrassment)	5	5	10
Female provider not available to do the test	0	1	1
Had personal or family responsibilities	4	1	5
Language/communication problems with provider	2	0	2
My doctor didn't think it was necessary	1	1	2
Transportation problems	1	0	1
Wait time was too long	1	1	2
Other, please specify	9	3	12

Participants in both groups were then asked why they had never done a Pap test or why they had not done one in the past 4 years (i.e. overdue). Participants could have chosen multiple responses from a list of pre-defined reasons and could also provide additional reasons that were not listed. For those who had never had a Pap test before, the most common reasons across both cohorts were that they ‘didn’t think it was necessary’ and that they ‘don’t know what a Pap test is.’ Many had also selected ‘other’ and provided a range of responses including lack of a provider, shyness/discomfort and not being advised about a Pap test in their home country, prior to coming to Canada. For participants who had done a Pap test before but were now overdue, ‘personal or family responsibilities’ was the most common reason for not doing a Pap test in the past 4 years across both cohorts, followed by ‘didn’t think it was necessary’ and ‘fear.’ Many also chose ‘other’ reasons, and these included: lack of reminder, time, access to a doctor, and previous discomfort with experience.

### Effectiveness

#### Cohort A

Of the 69 women who elected to try the self-sampling kit, 64 followed through, while 5 did not. Of the 5 that did not try it, 2 reported they subsequently did a Pap test, 1 changed their mind, and 2 were unreachable after exhausting all contact attempts. Of the 64 that did try the kit, 61 mailed it in and 3 did not. Of the 61 participants that mailed it in, 56 tested negative (i.e. HPV was not detected), 1 tested positive (i.e. HPV was detected), and 5 participants had their test come back as invalid/indeterminate. Whenever a test came back invalid/indeterminate, participants were contacted and offered a new self-sampling kit and their most recent test result is reported here. Of these 5, only 1 participant chose to re-do the self-sample and followed through with sending it to the lab for testing.

Of the 64 who tried the kit, 61 participants completed follow-up questions with the study coordinator or a community champion about their experience with the self-sampling kit. All 61 participants said they found the kit ‘user friendly’, and when asked if they found the instructions easy to follow, 57 said ‘yes’. 58 participants said they would do self-sampling again if it were offered to them. The 3 participants who said ‘no’ stated they would want to know the results of the self-sampling test first. Of the 61 participants who tried the HPV self-sampling kit, 44 of them had done a Pap test at some point in their life. Of the 44, 42 participants said they preferred HPV self-sampling over a Pap test.

#### Cohort B

Of the 39 people who elected not to try a HPV self-sampling kit (Cohort B), we successfully contacted and followed up with 38 of them, at least 3 months after their recruitment into the study. We were primarily interested in understanding if their participation in the study and interaction with the community champions, had an impact on their immediate decisions to get a Pap test. Of the 38 people we were able to get in contact with, 9 had gone on to get a Pap test and 28 did not. Of these 28, 19 had plans to get one in the near future, 5 did not and 4 were unsure.

#### Acceptability

In open-ended questions, participants in Cohort A were asked ‘why did you decide to use the kit?’ Ease of use, physical comfort, privacy and convenience were the most common responses. As one participant stated “We can do it at home, privacy issue is resolved, saves time as no need to wait for appointments or go to hospitals.” Another participant described self-sampling with “there is more privacy. I think it is not as invasive. There is more control—I can stop if I feel any discomfort. The size is small, so it will be less painful.” Another participant described her curiosity over this method being a ‘solution,’ saying “I would like to find a solution that can be done in the privacy of my home, does not require an appointment, and does not require travel and is convenient.”

Some cited their interaction with the community champion as being a reason, describing that ‘…I can do it on my own because [VP] explained well.’ Others were also convinced by family and friends who were also in the study. As one participant explains:

my sister informed me and we're both in the same situation where we are uncomfortable to go to the doctor. She told me about it and was very excited because you can do it at home. I don’t want to go to my own family doctor, and I know I need to get it done. Also wants to support research.

Lastly, many discussed simply wanting to get screened and to ease their mind. As one participant described her reasons for trying the kit: ‘want to find out about HPV and don't have time to go to the doctor. Want to make sure I’m safe and healthy and not in any kind of risk. Friend convinced me to do it. I'm having an open mind about it.’

#### Lack of acceptability

In contrast, participants in Cohort B were asked “why did you decide not to use the kit?” Many discussed their fears over screening and self-sampling. For some it was fear of the unknown ‘[I] have never done a Pap so I didn’t know what it involved. It was a fear of doing something I've never done.’ For other it was fear that it could be uncomfortable ‘I was little uncomfortable with the thought of any pain, I have a low threshold for pain, even blood works make me uncomfortable.’ One participant also cited fear of how her husband would react, as she described ‘My spouse would not like me to do anything without his permission. If I did this, it would be an issue for my future. If I have some disease, he will say I have lady with illness.’ Anecdotally, this was something we heard from some women during recruitment, when they declined to participate.

Others were apprehensive to try the kit because of prior healthcare experiences or medical issues. Fertility issues and potentially being pregnant were some reasons that were cited by participants. One participant discussed past health issues saying ‘I have some medical problems related to my private parts like pain, burning and discharge etcetera so I want to consult my doctor first.’

Some participants reiterated a lack of concern or conviction that screening was necessary, while others believed their sexual history (e.g. one partner, not being sexually active in the past 20 years) meant they no longer ‘needed’ to be screened.

Lastly, many in Cohort B also spoke of a lack of confidence to try the kit and a greater trust in their healthcare provider to perform such a test. For example, a participant said ‘I prefer going to a doctor to get my Pap done. I trust doctor more than myself.’ Another participant said ‘because it requires some precaution for hand hygiene I think, I would rather have a professional do it,’ while one participant went as far as thinking she may hurt herself ‘I don’t feel safe as I might not do it right and might hurt myself.’

## Discussion

The purpose of our study was to understand the reach and effectiveness of an HPV self-sampling intervention as an alternative to a Pap test for cervical screening, amongst a group of historically under screened (UNS) women in Ontario, Canada. Overall, we successfully utilized a community-based recruitment strategy that was led by community champions to reach over 1600 women. This strategy helped us to find 202 women that were eligible, of which 69 were interested in trying self-sampling. While some declined to do self-sampling because they were concerned about participating in research, others expressed how their fear and shyness were preventing them from being screened. While our study demonstrates that there is interest and acceptability of self-sampling as an alternative, it also shows us that self-sampling does not address all barriers and concerns to cervical screening like stigma surrounding sexually transmitted infections (STI) and other solutions are needed, as most women still chose not to participate. Similarly, in their study with women experiencing homelessness or housing instability, Ogilvie et al. [41] found only around 50% of those that were invited to participate, did so. Future programs should address these barriers that prevent women from getting an HPV test or Pap test.

Almost all of the women who elected to try the kit, followed through and mailed it in and successfully received results. During follow-up, all participants who had used the kit, said it was user-friendly, suggesting the kit to be acceptable to those who tried it. However, our findings provide guidance for ways to make self-sampling more successful. Some participants had to re-do the kit, which suggests that clearer instruction and/or more support once at home is needed. For most of the participants in Cohort B, their choice to not use the self-sampling device despite being UNS, was more an issue of fear and discomfort over screening and Pap tests, as well as the ‘unknown’ of this new device. Additionally, misconceptions and misinformation about cervical cancer risk was leading some to decide not to get screened at all, because they felt it was unnecessary. This suggests that for self-sampling to be successful amongst some UNS women, we must first address the misinformation and misconceptions that lead them to believe screening is unnecessary.

Furthermore, the role of family members in cervical screening also appeared to discourage screening for some women, suggesting that engagement is also needed for the family and friends of UNS women. In particular, engaging male partners or family members seems to be critical to the decision-making process, as some women approached for our study had cited needing their male family members’ permission. This was similarly found in a study of HPV self-sampling acceptability in Kenya, where some women who experienced opposition to screening by male partners, discussed anticipated negative reactions, lack of permission, and abandonment, while those that experienced support did so in the form of transportation, emotional support and encouragement [52]. The involvement of male partners in preventative and sexual health care has been effective in women’s health outcomes and is also found to be generally acceptable to women [52–56]. This is further emphasized by the World Health Organization that recommend the inclusion of family members and particularly male partners when conveying health education messages, is critical to the acceptance of screening services [56].

Although almost all participants understood the purpose of a Pap test, Pap test initiation, intervals and eligibility was less understood, suggesting that there is ample opportunity for education on current screening guidelines in the province, particularly where there may be confusion with differing guidelines in other provinces and countries. Some participants also expressed being forgetful or needing reminders for when they are due for a Pap test. HPV self-sampling kits can also be useful here, as women who are overdue can be directly mailed a kit. This is a strategy already being used in countries such as Australia, that have switched from Pap test to HPV tests [57].

Questions on attitudes towards cervical screening and Pap tests in particular, showed that privacy, comfort and time were a large concern. These barriers were similarly seen when participants were asked why they were overdue for screening. HPV self-sampling begins to address these barriers as women can collect the sample on their own, outside a healthcare space (i.e. their home), and during a time where it is convenient for them. More people in Cohort A were concerned with privacy during a Pap test, and this could explain why they chose to try self-sampling compared to those in Cohort B who were less concerned. During follow-up, almost all participants confirmed that self-sampling was their preferred method and highlighted the privacy and comfort of the device for cervical screening. This suggests that HPV self-sampling would be acceptable over the Pap test for women concerned with privacy and comfort, and that the HPV self-sampling device also was perceived as less invasive than a Pap test. Concern over confidence to collect the sample, however, still needs to be addressed. Some found the community champion and knowing previous participants as being critical for confidence to try the device. Effective knowledge translation in the form of visuals or peer support to explain the device, will be needed to successfully implement the device as an alternative to a Pap test. Additionally, these devices should have multiple ways to access them, as not everyone has access to a healthcare provider or feels comfortable having this discussion. Being able to access a kit in places in the community will be important to engage more people in screening.

As well, the COVID-19 pandemic shed additional light on the utility of mail-in HPV self-sampling devices. We saw firsthand how participants were able to still complete their screening using these devices, even when Pap tests were put on a pause in the province. Participants were able to receive the kits via mail and return them to the lab to be tested. This also provides an alternative to people who may usually engage in Pap tests but in the context of a pandemic are hesitant to move around in the community or visit a healthcare provider. In the coming months and potentially years, as the province and other jurisdictions work through the backlog of cervical screening for people who missed their Pap test during the pandemic, HPV self-sampling devices may be a useful approach to getting people screened.

It is important to highlight that while self-sampling can improve participation in screening, the study data are insufficient to say that HPV self-sampling can replace clinical cervical cancer screening completely. Screening is only valid for asymptomatic cases and those that are symptomatic would need further clinical examination. There are some potential disadvantages in HPV self-sampling and that can include decreased engagement with healthcare providers that can lead to missed opportunities to address STI screening or health in general. If the HPV self-sample result is abnormal we would want to know that people are willing to see a clinician for follow-up testing, which may see some of the same barriers that have lead to underscreening (e.g. privacy concerns, discomfort, access, etc.).

### Strengths

This study demonstrates the utility of community champions in cervical screening and encouraging uptake, even beyond a research study. For women who elected to try the kit, the confidence they got from their conversation with the community champion and/or peers that had previously participated, were highlighted, suggesting the important role community champions and peers have in the acceptability of the device. Our community champions engaged with the community and built on existing relationships to advertise the study and approach potential participants. The relatedness of community champions to women in the study also helped address some of the discomfort women in this study had, to discuss topics of sexual health, cancer screening and healthcare interactions. For example, emotion-laden responses such as fear, anxiety and shyness around getting a Pap test have been found to prevent many South Asian women from being screened [58–65].

Community champions were particularly effective in engaging women outside of healthcare spaces, including social and entertainment events, neighborhood associations, tea parties and word-of-mouth. This meant we were able to engage women who may not be accessing healthcare, and taking uncomfortable conversations into more casual and familiar environments. The relatedness of the community champions (e.g. age, gender, ethnicity, immigration experience, language) was effective in creating trust and comfort to try HPV self-sampling, and this was particularly important for building confidence to try the device. Additionally, the approach was effective amongst some that chose not to try the kit, as it encouraged them to get screened via a Pap test with a healthcare provider. While successful, the community champion approach did face some institutional challenges early on. Traditionally, research ethics approvals do not let people known to participants interview them as they are concerned with potential coercion and privacy issues. In the early days of the study, some potential participants declined participating if the community champion they knew was not allowed to interview them. After lengthy discussion, we received ethics approval for our community champions to interview participants they knew, as long as the participant was comfortable with it. All participants had the option to do the interview with another community champion or the study coordinator.

Additionally, the majority of our recruitment occurred in the community and through word-of-mouth, in spaces that are not always used for healthcare. This allowed us to recruit people who may not have been accessing healthcare spaces.

### Limitations

This study has several limitations of note. Due to limited study funds, our study focused recruitment efforts in Peel Region and the demographics and size of our final sample reflected largely that. The majority of our participants were South Asian, particularly identifying as Indian or Pakistani. This is reflective of both the demographics of Peel as well as the community champions themselves. Between our community champions, we were able to provide language assistance in Hindi, Marathi, Gujarati, Punjabi, and Urdu. While this allowed us to recruit many more women than an English-only study, it is not fully representative of all the languages spoken in South or West Asia, the Middle East and North Africa, so our recruitment was limited and our sample size was small. It is also important to highlight the diversity along the lines of religion, ethnicity, social class, and age, to name some. While our community champions were able to gain access to many different locations and groups, their relatedness and peer status varied with some participants relating more to them than others. Future research and screening programs should employ several community champions that reflect this diversity of South Asian, West Asian, Middle Eastern and North African communities. Our study focused on women and did not include their male partners who play an important role in decision making, Future studies should include male partners in sexual health education and explore the impact it may have on cervical cancer screening uptake.

Furthermore, misconception and stigmas surrounding sexually transmitted infections (STIs) including HPV may deter uptake of screening due to entrenched gender norms and stereotypes associated with infections (e.g. women with STIs may be viewed as immoral, corrupted, or other demeaning labels). Our study did not explore this component as a barrier to screening.

Lastly, we were limited by the confines of research as the ‘study’ aspect, including gathering data and signing consent, was a barrier for some women to participate in self-sampling or even trust the research team.

## Conclusion/next steps

This study has provided important insights into the reach and effectiveness of HPV self-sampling as an alternative to a Pap test for cervical screening for largely under- or never-screened women in Ontario, Canada. The study showed that many of these women had at least some knowledge of cervical cancer and Pap tests, but that the logistics, fear, comfort and privacy concerns were the most important barriers to tackle. HPV self-sampling addresses many of these concerns as women can use a take-home kit wherever they feel comfortable, and can privately collect the sample themselves.

We also showed the effectiveness of community champions in engaging women to talk about cervical cancer and screening, and to also encourage them to get screened. Whether they chose and followed through with the self-sampling device, or went on to get a Pap test after engaging with the study, our community champions demonstrated the need and impact on people when they have someone they feel comfortable and safe to speak with.

Our next steps are to analyze and present the rich qualitative data that dives further into cervical cancer screening amongst these groups of women and what they may or may not like about HPV self-sampling. Through focus groups and one-on-one interviews, we were able to collect data on people’s reasons for under screening or never screened, as well as their thoughts and experiences with HPV self-sampling. This will be valuable information as the province of Ontario will soon move to HPV testing to replace Pap tests, and the option for self-sampling will eventually become available.

## Data Availability

The datasets generated and/or analysed during the current study are not publicly available due to maintaining the privacy and confidentiality of participants, but are available from the corresponding author on reasonable request.

## Abbreviations

D&I: Dissemination and implementation
HPV: Human papillomavirus
OCSP: Ontario cervical screening
Pap Test: Papanicolaou test
PEBC: Program in evidence based care
RE-AIM: Reach, effectiveness, adoption, implementation, maintenance
SPSS: Statistical package for the social sciences
STI: Sexually transmitted infections
UNS: Under- or never-screened
